# Carbonate chemistry of an in-situ free-ocean CO_2_ enrichment experiment (antFOCE) in comparison to short term variation in Antarctic coastal waters

**DOI:** 10.1038/s41598-018-21029-1

**Published:** 2018-02-12

**Authors:** J. S. Stark, N. P. Roden, G. J. Johnstone, M. Milnes, J. G. Black, S. Whiteside, W. Kirkwood, K. Newbery, S. Stark, E. van Ooijen, B. Tilbrook, E. T. Peltzer, K. Berry, D. Roberts

**Affiliations:** 1 Antarctic Conservation and Management Theme, Australian Antarctic Division, 203 Channel Hwy, Kingston, 7050 Tasmania Australia; 2 Institute for Marine and Antarctic Studies, University of Tasmania, 20 Castray Esplanade, Hobart, Australia; 3 Monterey Bay Aquarium Research Institute, 7700 Sandholdt Rd, Moss Landing, CA 95039 United States of America; 4 CSIRO Oceans and Atmosphere, Castray Esplanade, Hobart, TAS 7000 Australia; 5 Antarctic Climate and Ecosystems Cooperative Research Centre, University of Tasmania, 20 Castray Esplanade, Hobart, Tasmania 7000 Australia

## Abstract

Free-ocean CO_2_ enrichment (FOCE) experiments have been deployed in marine ecosystems to manipulate carbonate system conditions to those predicted in future oceans. We investigated whether the pH/carbonate chemistry of extremely cold polar waters can be manipulated in an ecologically relevant way, to represent conditions under future atmospheric CO_2_ levels, in an *in-situ* FOCE experiment in Antarctica. We examined spatial and temporal variation in local ambient carbonate chemistry at hourly intervals at two sites between December and February and compared these with experimental conditions. We successfully maintained a mean pH offset in acidified benthic chambers of −0.38 (±0.07) from ambient for approximately 8 weeks. Local diel and seasonal fluctuations in ambient pH were duplicated in the FOCE system. Large temporal variability in acidified chambers resulted from system stoppages. The mean pH, Ω_arag_ and *f*CO_2_ values in the acidified chambers were 7.688 ± 0.079, 0.62 ± 0.13 and 912 ± 150 µatm, respectively. Variation in ambient pH appeared to be mainly driven by salinity and biological production and ranged from 8.019 to 8.192 with significant spatio-temporal variation. This experiment demonstrates the utility of FOCE systems to create conditions expected in future oceans that represent ecologically relevant variation, even under polar conditions.

## Introduction

The level of anthropogenic carbon dioxide (CO_2_) in the atmosphere continues to rise in line with the highest Intergovernmental Panel on Climate Change (IPCC) emissions scenario, representative concentration pathway (RCP) 8.5^1–3^. The oceans have absorbed approximately 28% of CO_2_ emitted since 1850^4^. This increase in oceanic CO_2_ causes an increase in the hydrogen ion concentration [H^+^] and has resulted in a decrease in seawater pH of 0.1^5^ and an accompanying decrease in the saturation state (Ω) of calcium carbonate (CaCO_3_). This process – known as ocean acidification – causes significant changes in the chemistry of seawater, particularly the carbonate system. Under IPCC emission scenarios for increasing CO_2_, ocean pH is predicted to decline by a further 0.2 to 0.4 by 2100^6^. This may have serious consequences for marine ecosystems^5^, from direct effects on physiology, metabolism, and availability of carbonate to build shells and skeletons for many species; to indirect effects on food webs and species interactions^7^.

Polar waters have naturally low carbonate ion concentrations, making them particularly susceptible to ocean acidification, due to a higher CO_2_ solubility in cold water, sensitivity of acid-base disassociation coefficients, and oceanic circulation^8,9^. The Southern Ocean is predicted to be one of the first regions to experience widespread undersaturation of aragonite, a major biogenic form of CaCO_3_, by 2050^5^ or as early as 2030 when seasonal variation of carbonate chemistry is considered^10^. Undersaturation in Arctic seas could occur as early as 2020^11^. Dissolution of aragonitic shells has already been observed in pelagic pteropods in the Southern Ocean^12^. These changes may have serious consequences for many calcifying organisms in polar regions and alter the structure and biodiversity of polar ecosystems at multiple trophic levels^5,13^. There is currently a relative paucity of knowledge of the effects of ocean acidification on polar marine organisms and ecosystems in comparison to other latitudes^8^. There are also relatively few high resolution or long-term observations of natural spatial or temporal variation of carbonate chemistry in Antarctic waters.

Free-ocean CO_2_ enrichment (FOCE) systems were developed to address the need for *in-situ* studies of the effects of ocean acidification over long time scales^14^. Other *in-situ* methods for investigating ocean acidification, such as natural analogue CO_2_ vents, are complicated by factors outside of the experimenters’ control such as regulation of treatment conditions, selection of reference sites, large temporal variability^15^, uncontrolled environmental changes and migration of organisms into and out of the study areas^14^. FOCE systems allow for the precise control of pH (by CO_2_ enrichment) as an offset from ambient pH, whilst allowing for natural variation in other environmental parameters; and for long term experiments (from weeks to months) on intact communities, including examination of indirect effects such as species interactions and food webs^14^. They provide a crucial link between organism level studies and scaling up to ecosystem effects^16,17^.

Carbonate chemistry of coastal systems can be highly variable on timescales from days to months, with multiple drivers including freshwater input, primary productivity and seasonal upwelling^18,19^. The interaction between biological and geochemical processes in coastal ecosystems has the potential to either exacerbate or mitigate ocean acidification effects^19^. The frequency, amplitude and duration of carbonate chemistry events that cross ecological thresholds will be crucial to understanding effects of ocean acidification on coastal organisms^19^. Future changes in Antarctic coastal carbonate chemistry will be influenced by changes in sea ice, stratification and mixing, freshwater inputs^20,21^ and biological processes^18^. Observations of pH from Antarctic coastal waters are sparse and very limited, but have been shown in some areas to have variability of a similar magnitude to kelp forests and coral reefs^22–24^. There is a critical need for high resolution records of pH in Antarctic coastal waters to understand current conditions, variability, regional differences and how these areas may respond to future change.

FOCE systems were developed at the Monterey Bay Aquarium Research Institute (MBARI)^25,26^ and have been deployed in various locations around the world^14^. FOCE systems consist of semi-open flow-through chambers enclosing a section of the seabed, into which a small quantity of CO_2_ enriched seawater is introduced, usually via a duct or flume to allow equilibration of pH before exposure inside the chambers. The manipulation of pH is usually done as an offset^27,28^ from ambient conditions, incorporating natural variation in pH in the acidified treatments. They are semi-open systems in that the primary water source comes from outside the chambers, incorporating natural variation in background physicochemical variables such as temperature, oxygen and salinity, as well as biogeochemical and biological variables such as nutrients, food and propagules. The incorporation of natural variation into the FOCE experiment is one of the most important features in creating realistic experimental ‘future ocean’ treatments. There have been no previous attempts to manipulate pH in polar waters using these methods, where the reaction kinetics of CO_2_ in seawater are strongly influenced by low temperatures^29^. *In situ* manipulation of seawater carbonate chemistry requires sufficient periods of equilibration to stabilise experimental conditions^14,29^.

Here we investigate whether it is possible in polar waters to create ecologically meaningful pH conditions predicted in future oceans using FOCE technology. We also examined the variation in pH and other parameters at a coastal Antarctic site over a 3 month period between December and February at a high temporal resolution of hourly measurements. We describe the performance of the antFOCE system and report on the changes in carbonate chemistry that were observed during the experiment, in relation to observations of ambient pH at the experimental site, O’Brien Bay, Casey Research Station, East Antarctica. We aimed to achieve a pH perturbation of −0.4 from ambient, in line with IPCC predictions under business as usual emissions scenarios for the year 2100, which equates to atmospheric CO_2_ levels of approximately 936 ppm (IPCC AR4, SRES A1FI, and AR5 RCP8.5)^30^. By maintaining a pH offset of −0.4 and allowing for natural pH variation, the pH in the acidified chambers would vary according to diel, tidal, seasonal and other factors that influence pH in coastal waters.

## Results

### Manipulation of the carbonate system and pH

**Figure 1:**
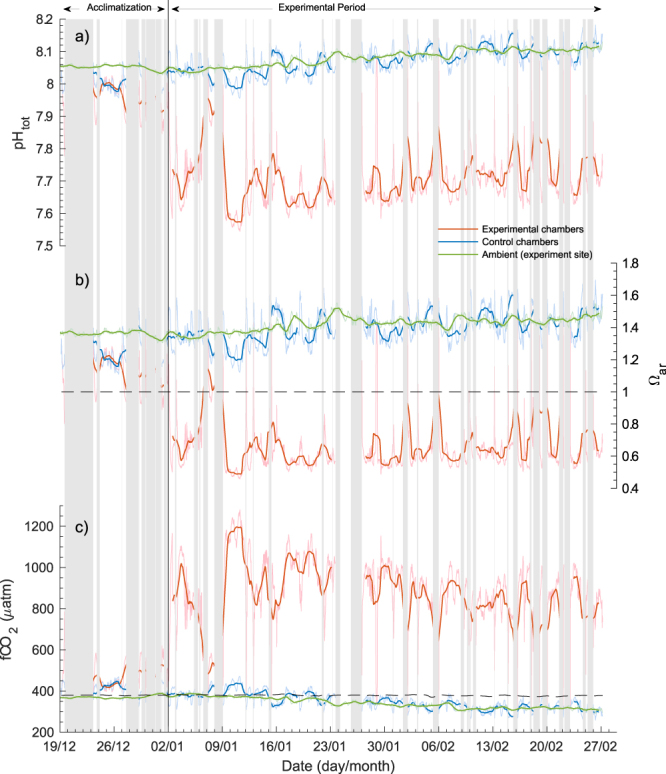
Carbonate chemistry conditions within the experimental chambers and in ambient seawater during the acclimatization and experimental periods of the antFOCE experiment. (**a**) Measured pH_tot_; (**b**) calculated saturation state of aragonite (Ω_ar_); (**c**) calculated fugacity of CO_2_ (*f*CO_2_) (µatm) of seawater with atmospheric *f*CO_2_ shown as dashed line. Green line = ambient conditions at experiment site; red line = acidified chambers (mean of chambers A and B); blue line = control chambers (chambers C and D combined). Lighter coloured lines show high-resolution measurements (taken every 30 min), with darker coloured lines showing 24 hour low-pass (moving average) filtered data. The grey bars represent periods of experimental system downtime.

The biogeochemical parameters in the antFOCE system, measured from 19 December 2014 to 27 February 2015, are summarized in Fig. 1 and weekly averages shown in Supplementary Table S1. The antFOCE deployment period is divided into two separate phases, a two-week acclimatization phase and an eight-week experimental phase. During the acclimatization phase, the pH of the acidified chambers was gradually lowered by incrementally increasing the amount of CO_2_ Enriched Seawater (ESW) introduced into the chambers until the target pH reduction of 0.4 relative to the control chambers was reached. During the eight-week experimental phase, a mean pH offset of −0.383 ± 0.066 and −0.382 ± 0.065 was achieved for chambers A and B respectively. This resulted in aragonite undersaturation, with mean Ω_ar_ values of 0.62 ± 0.15 and 0.62 ± 0.13 and mean *f*CO_2_ values of 914 ± 159 µatm and 911 ± 150 µatm (Supplementary Table S1) in the acidified chambers. The control chambers during the experimental phase had a mean Ω_ar_ of 1.39 ± 0.11. The mean *f*CO_2_ in the control chambers was 354 ± 42 µatm and in the ambient environment was 341 ± 25 µatm, which was undersaturated with respect to the mean atmospheric *f*CO_2_ value of 379 ± 3 µatm (1 s.d.).

**Figure 2:**
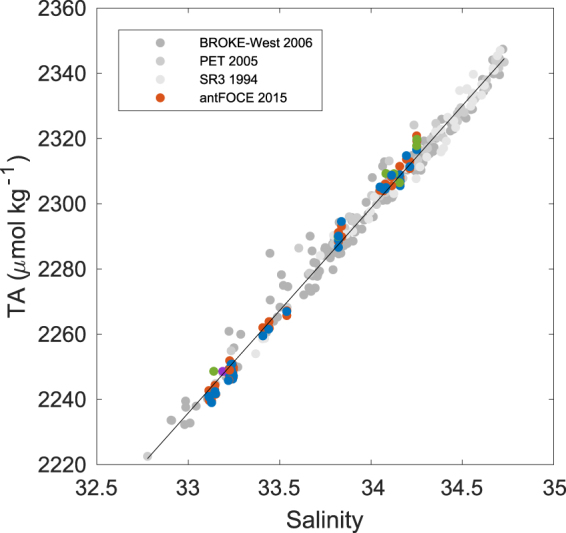
The salinity and TA relationship in waters south of 60°S and shallower than 500 m, including the antFOCE experiment. Grey dots show data from BROKE-West in 2006, the Princess Elizabeth Trough in 2005 (PET) and the WOCE SR3 transect in 1994 (data available from GLODAPv2). Coloured dots show values measured in this study (red = acidified chambers, blue = control chambers, green = ambient seawater at experiment site, purple = ambient seawater at outer O’Brien Bay). A linear regression yielded the following equation, y = (62.8 ± 0.4)x + (163 ± 13) (n = 340, r^2^ = 0.99, standard error = 4 μmol kg^−1^).

A comparison of measured Total Alkalinity (TA) values in the acidified and control chambers showed no detectable increase as a result of CaCO_3_ dissolution (Fig. 2), despite the acidified chambers having undersaturated Ω_ar_. This was initially determined by normalising (*n*) the sum of TA and nitrate (TA + N) concentrations to the mean salinity of 33.645 observed during the antFOCE deployment period. This accounts for changes in these parameters as a result of freshwater input/removal, and summing the concentrations of TA and nitrate accounts for changes in TA associated with the uptake or release of dissolved nitrate during photosynthesis or respiration^31^. Consequently, both the acidified and control chambers had similar mean *n*TA + *n*N values of 2302 ± 3 µmol kg^−1^ (1 s.d.; n = 57) and 2301 ± 3 µmol kg^−1^ (1 s.d.; n = 28), respectively. This result is not unexpected given the short residence time of water within the chambers of ~67 seconds. If we assume a maximum dissolution rate^32^, porosity and carbonate content within the sediments (<10%, actual values range from 1–3%), the expected flux of alkalinity due to CaCO_3_ dissolution equates to only 0.0009 µmol L^−1^, which would be undetectable given the accuracy and precision of the TA measurements of ±1.3 µmol kg^−1^ (see methods).

During the acclimatization and experimental phases, the system experienced a series of power outages and stoppages due to equipment failure. The failure of the onsite power generator, due to a faulty oil pressure sensor, caused the majority of these outages except for two occasions, where the failure of individual thrusters, drawing water through the chambers, resulted in system stoppages. Divers replaced the damaged thrusters and the system was restarted within 24 hours. During these system stoppages, CO_2_ system parameters within the acidified chambers would slowly revert back to ambient conditions until the system could be restarted. Because measuring the pH of the chambers relied on an external power supply, we were unable to characterize the biogeochemical conditions within the chambers during these short periods. Therefore, the results we report here represent the conditions within the antFOCE chambers from when the system was fully operational. Upon restarting the experimental system the pH was gradually brought back to the desired offset from ambient. This would typically be achieved over a 2 to 4 hour period. Over the 14 day acclimatization period, 8.55 days were lost due to system stoppages, and over the 56 day experimental period, 12.19 days were lost due to system stoppages (downtime), as indicated by the grey shaded areas on Fig. 1 and summarized in Supplementary Table S1.

**Figure 3:**
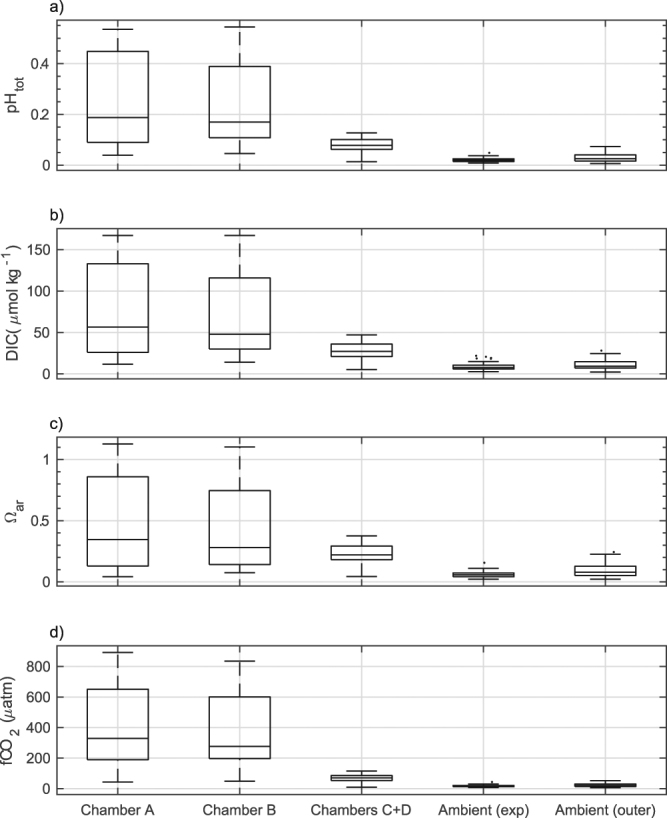
Summary of daily changes in seawater parameters within O’Brien Bay over the duration of the experiment. Boxes show the interquartile and median daily range. The whisker length is 1.5x the interquartile range. Dots show data points that lie outside of that range. Data shown are of (**a**) pH total scale (pH_tot_), (**b**) calculated dissolved inorganic carbon (DIC), (**c**) saturation state of aragonite (Ω_ar_) and (**d**) fugacity of CO_2_ (*f*CO_2_). Chambers A and B are the acidified chambers; chambers C+D are the combined values of the control chambers; Ambient (exp) shows the natural conditions at the site of the experiment; Ambient (outer) shows conditions at the outer site in O’Brien Bay.

These system stoppages resulted in increased variability within the chambers (Fig. 3) and in particular within the acidified chambers, which had a mean pH diel variability of 0.24 ± 0.17 in comparison to a mean of 0.077 ± 0.027 in the controls and a mean of 0.020 ± 0.008 in ambient seawater at the site (Supplementary Table S2). The mean values of carbonate system parameters within the control chambers throughout the experimental period, when considered over weekly intervals, were similar to those observed in the ambient environment (Fig. 1, Supplementary Table S1).

### High resolution ambient variability

**Figure 4:**
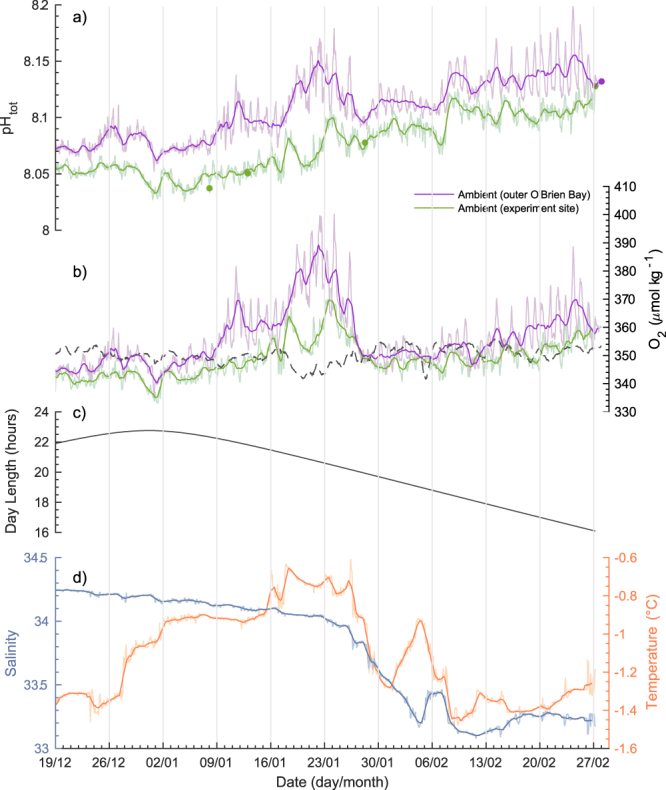
Ambient seawater conditions at two sites in O’Brien Bay, Casey Research Station, Antarctica, the site of the antFOCE experiment. Measurements were made between 19 December 2014 and 28 February 2015. (**a**) pH_tot_ (dots show calibration points where discrete samples were taken for analysis); (**b**) dissolved oxygen (O_2_) with oxygen saturation shown as dashed line; (**c**) day length at O’Brien Bay (hours); (**d**) salinity and seawater temperature from the experiment site. Lighter coloured lines show high-resolution (hourly) measurements, with darker coloured lines showing 24-hour low-pass (moving average) filtered data.

**Figure 5:**
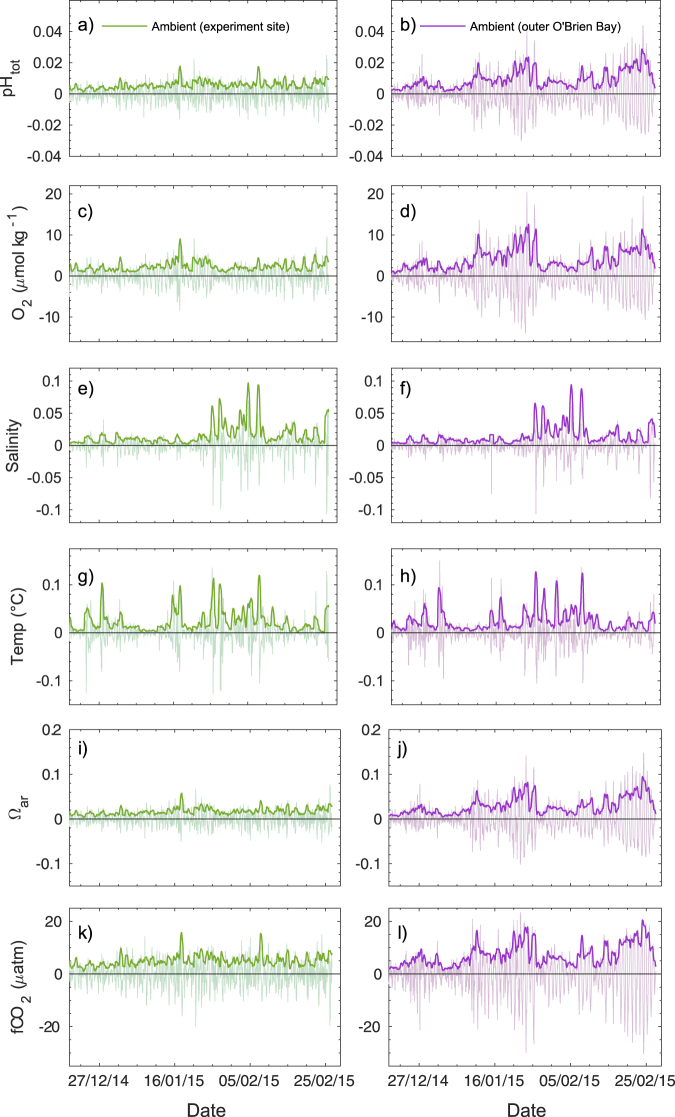
Short-term variability (diel changes) in ambient seawater conditions at two sites in O’Brien Bay, Casey station Antarctica. High pass filtered data showing: (**a**) and (**b**) pH_tot_; (**c**) and (**d**) dissolved oxygen (µmol kg^−1^); (**e**) and (**f**) salinity; (**g**) and (**h**) temperature (°C); (**i**) and (**j**) aragonite saturation state; (**k**) and (**l**) fugacity of CO_2_. The zero represents a moving 24-hour average value at any given time. Darker coloured lines are the standard deviation of the 24-hour moving average of the high-frequency data (lighter coloured lines).

**Figure 6:**
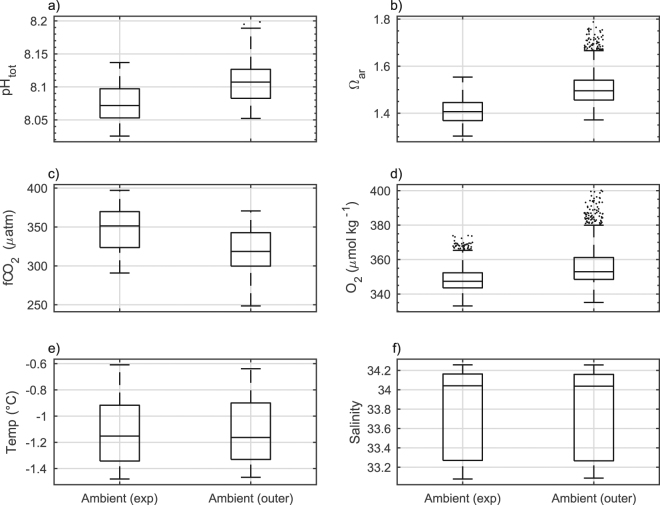
Summary of summer ambient seawater conditions within O’Brien Bay, the site of the antFOCE experiment between 19 December 2014 and 27 February 2015. Boxes show the interquartile range and median values. The whisker length is 1.5x the interquartile range. Dots show data points that lie outside of that range. Data shown are of (**a**) pH total scale (pH_tot_), (**b**) saturation state of aragonite (Ω_ar_), (**c**) fugacity of CO_2_ (*f*CO_2_), (**d**) dissolved oxygen (O_2_), (**e**) temperature and (**f**) salinity. Ambient (exp) shows the natural conditions at the site of the experiment; Ambient (outer) shows conditions at the outer site in O’Brien Bay.

There was a significant linear increase in observed ambient pH at both sites during the three month period (Experiment site R^2^ = 0.75, p < 0.0001; Outer site R^2^ = 0.67, p < 0.0001) with a seasonal pH range of 8.025 to 8.137 at the experimental site and 8.052 to 8.198 at the outer site (Fig. 4a, Supplementary Table S2b). Oxygen concentration at both sites peaked in late-January (Fig. 4b), just before a significant decrease in salinity was observed throughout the bay (Fig. 4d) due to an influx of glacial melt water. Temperature and salinity were very similar at both sites (Fig. 5e–h; 6e,f). Ambient pH and oxygen concentration were generally higher at the outer O’Brien Bay site than at the experiment site (Supplementary Table S2 and Figs 4 and 6).

In comparison to the experiment site (Fig. 5a), the outer site experienced an increase in short-term (diel) pH variability (Fig. 5b) over the deployment period. The range in dissolved oxygen concentrations over the duration of the experiment was also smaller at the experiment site (Figs 5c, 6d) compared to the outer site (Figs 5d, 6d). The aragonite saturation state and *f*CO_2_ were also more variable at the outer site (Fig. 5i–l). Sea-ice cover of different thickness and age characterized these two locations, with multi-year sea ice over the experimental site measuring 260 cm in thickness, and first-year ice over the outer O’Brien Bay site measuring 150 cm in thickness at the start of the deployment.

There were significant differences in pH at both sites at all temporal scales examined: month, week, day, diel and hour (PERMANOVA analysis, Table 1). The relative magnitude of the effect size of each scale indicated that, as expected, the largest differences were between different months. Variation in pH at hourly (residual), diel, and daily intervals were of the same magnitude at the inner site, but at the outer site the diel variation had a much great effect size than daily variation (Table 1).

**Table 1:** PERMANOVA table of results for temporal differences in pH at nested hierarchical scales.

Experiment site						Outer site				
Source	Df	MS	Pseudo-F	P	% var	Df	MS	Pseudo -F	P	% var
Month	2	0.319	13.4	0.001	70	2	0.290	9.2	0.002	54
Week (Month)	9	0.028	33.6	0.001	22	9	0.036	14.2	0.001	24
Day (Wk(Mth))	62	0.0008	2.9	0.001	3	62	0.003	2.1	0.003	6
Diel (D(W(M)))	73	0.0003	11.1	0.001	3	74	0.001	20.7	0.001	10
Hour (Residual)	1605	0.00003			3	1622	0.00006			6
Total	1751					1769				

% var = Estimates of components of variation of pH at each temporal scale.

The dissolved oxygen concentration was, on average, 8 µmol kg^−1^ higher and the calculated DIC concentration ~13 µmol kg^−1^ lower at the outer site (Supplementary Table S2). Dividing the difference in oxygen concentration by 1.4 allows this value to be expressed in terms of organic carbon production, or DIC uptake^33,34^. Therefore, for a photosynthetic oxygen production of 8 µmol kg^−1^, an approximate 6 µmol kg^−1^ of DIC uptake would be expected. This explains the majority of the offset observed between the two sites when the uncertainty of calculated DIC concentrations (±4 µmol kg^−1^) is considered. However, when biological changes between the two sites are calculated over daily intervals, the outer site was not significantly different from the experimental site (Supplementary Fig. S3). This is highlighted by the mean daily values of net community production (NCP; *NCP* = *Net Primary Production – Heterotrophic Respiration*) of 3 ± 38 mmol C m^−2^ day^−1^ (1 s.d.; 73 days) at the experimental site (range of −68 to 131 mmol C m^−2^ day^−1^) and 2 ± 48 mmol C m^−2^ day^−1^ (1 s.d.) at the outer site (range of −142 to 101 mmol C m^−2^ day^−1^). Other contributors to daily changes in DIC, namely air-sea flux and ice formation and melt, also show similar contributions between each site (Supplementary Fig. S3). The largest changes in DIC were caused by freshwater input from ice melt, observed in late January and early February. Even though seawater *f*CO_2_ was mostly undersaturated with respect to the atmosphere, 100% sea ice cover for the duration of the study caused the air-sea CO_2_ flux to be near zero. NCP was however, extremely variable on short timescales, switching from net production to net heterotrophy from day to day (Supplementary Fig. S3).

Distance based linear modelling (DISTLM^35^), using permutation tests^36^, was used to investigate correlative relationships between pH and other variables. This indicated that a significant proportion of the variation in pH in models could be explained individually by either salinity, oxygen or temperature, but the amount of variation explained differed between the experiment site and the outer site (Table 2). Salinity could account for 80% of the variation in pH at the experiment site, followed by oxygen (37%). Salinity and oxygen explained similar levels of variation in pH at the outer site, but temperature had only a very weak relationship with pH (Table 2). Oxygen accounted for a much higher percentage of pH variation at the outer site than the experiment site, corresponding to greater variation in oxygen (Fig. 5c,d) and NCP (Supplementary Fig. S3) at the outer site. Linear models containing all three variables (salinity, oxygen and temperature) explained approximately 95% of the variation in pH at both sites (Table 2).

**Table 2:** Results of distance based linear modelling (DistLM) analysis of pH using salinity, temperature and oxygen concentration as predictor variables.

Experiment site					Outer site			
Marginal test								
Variable	SS (trace)	Pseudo-F	P	Prop.	SS (trace)	Pseudo-F	P	Prop.
Oxygen	0.41	1019.5	0.001	0.37	0.63	1574.4	0.001	0.47
Temperature	0.19	365.48	0.001	0.17	0.02	27.607	0.001	0.02
Salinity	0.88	6973.3	0.001	0.80	0.71	1991.3	0.001	0.53
	R^2^	RSS			R^2^	RSS		
Combined model	0.96	0.040			0.95	0.064		

Prop. = proportion of variance explained; RSS = residual sums of squares. Combined model includes all three variables.

## Discussion

Unlike free-air CO_2_ experiments (FACE)^37^ where simple CO_2_ addition to the air is all that is required, the reaction of CO_2_ with water in seawater introduces a kinetic delay that is both time and temperature dependent^29^. Given the very slow kinetics in very cold Antarctic waters, in order to be completely free and open, a FOCE experiment would need a ring of CO_2_ emitters that would be eminently impractical both in size and CO_2_ requirements to ensure that a sufficiently large portion of the “enclosure” would experience equilibrium of the CO_2_ enrichment at maximum seawater velocities for the region. The flume design used in FOCE is thus a compromise between the ideal fully open system and one where the CO_2_ conditions are within the experimenters’ control. The antFOCE system successfully altered the *in-situ* carbonate chemistry in acidified chambers to conditions predicted in future oceans, maintaining a pH offset of −0.38 (±0.07). Conditions in control chambers closely matched background ambient fluctuations, but variation in acidified chambers was greater than that observed in the local ambient environment. There was considerable natural diel variation, however, towards the end of the deployment period in late February. The antFOCE system experienced several technical challenges including thruster (flow regulator) failure and problems with maintaining a reliable power supply in a remote location, which altered the experimental manipulation of carbonate conditions. Power outages were attributed, post-experiment, to a faulty oil pressure sensor in the power generator, and were the main technical issue experienced during the experiment. These problems could largely be avoided in future FOCE designs through the implementation of backup power systems. Aspects of the system that worked well included the surface housed sensor system, making water sampling and maintenance of sensors convenient and straightforward. The Honeywell Durafet pH sensors proved to be highly reliable in monitoring pH in both the experimental system and in the ambient environment.

### Manipulation of carbonate chemistry in relation to natural variation

**Figure 7:**
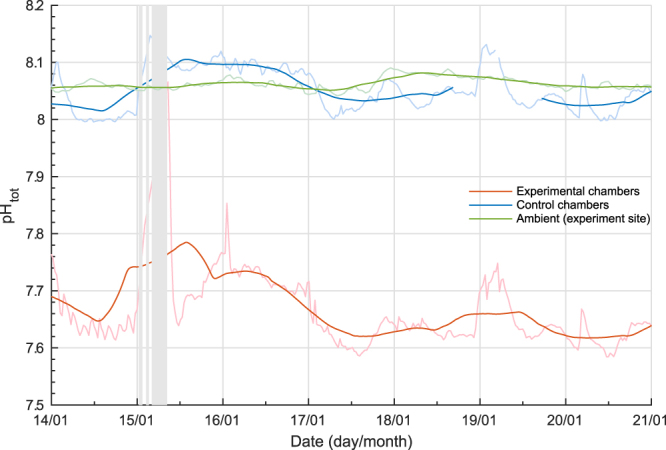
High-resolution record of pH in the antFOCE experiment and in the ambient seawater over a 7-day period. Measurements of pH_tot_ taken between 14 January 2015 and 21 January 2015. Green line = ambient pH at experiment site; red line = pH of acidified chambers (mean of chambers A and B); blue line = pH of control chambers (chambers C and D combined). Lighter coloured lines show high-resolution measurements, with darker coloured lines showing 24-hour low-pass (moving average) filtered data. The grey bars represent periods of experimental system downtime.

Conditions in the acidified chambers represented a significant change from conditions likely to be experienced over a full annual cycle in Antarctica^20,23,24,38,39^. During the experimental phase, mean pH in the acidified chambers was 7.688 ± 0.079, the mean saturation state of aragonite was 0.62 ± 0.13 and the mean *f*CO_2_ was 912 ± 150 µatm. These conditions are within the range predicted under the IPCC RCP8.5 “business as usual” scenario by 2100^30^. The antFOCE system, in line with xFOCE goals^14^, altered and maintained a pH offset in the acidified chambers that closely tracked the diel variation observed in the control chambers (Fig. 7), when the system was operating normally. System outages, however, resulted in periods of diel variability in the acidified chambers (mean range of 0.24 ± 0.17) that were much larger than the control chambers or ambient conditions (Supplementary Table S2). The pH reverted fully or partially back to ambient during outages (Supplementary Table S1).

High resolution observations of pH are sparse in Antarctic coastal waters, but where available they indicate short term (daily to weekly) variation to be as high as 0.13 over the course of hours and up to 0.27 over several days in McMurdo Sound^23,40^; and up to 0.13 on the Antarctic Peninsula^24^. Thus the increased variability in acidified chambers is not outside the range of natural fluctuations currently experienced in some coastal Antarctic regions. In a global context the large diel variability in acidified treatments was not as extreme as the short-term pH variability (days to weeks) observed in other coastal ecosystems^22^, including kelp forests (range: 0.544), estuaries (range: 0.992), upwelling systems (range: 0.467) and CO_2_ vents (range: 1.43).

The large diel variation in pH and other carbonate parameters observed in the acidified treatments will need to be considered when interpreting the biological responses, but these large pH fluctuations may provide insights into the level of resilience of Antarctic benthic communities to rapid changes in environmental conditions. This is especially relevant as future pH variability is expected to increase with reduction in the buffer capacity of seawater as more CO_2_ is absorbed from the atmosphere, particularly in polar waters^8,41,42^. Understanding how diel and seasonal variability in dissolved CO_2_ parameters will change in response to future ocean acidification is of great interest to the biogeochemistry community and FOCE technology is a useful tool for investigating these changes under *in-situ* conditions of natural variation where multiple drivers of changes in pH occur.

The pH of the control chambers also had greater variability than that measured in the ambient environment. A similar observation was made in the European FOCE experiment (eFOCE)^28^ conducted on a seagrass meadow, where the median diel pH variation was 75% greater in the control chambers compared to the ambient environment. The mean diel pH variation in antFOCE control chambers was as much as 285% greater (Fig. 3, Supplementary Table S2). This variation could have been due to the influence of the chambers themselves on the enclosed biological communities, or alternatively, biological activity or gas exchange within the sample lines may have contributed to the observed changes. It is unlikely that other physical processes within the sample lines, such as warming, could be responsible for such variations in pH, as temperature changes caused by the heat trace in the sample lines (~3 °C of warming at the sensors) for example, were accounted for when adjusting measured pH values to *in-situ* conditions. The small volume of air that was occasionally observed in the sample hoses may have contaminated seawater sampled from the chambers, although this volume of air was very small when compared to the volume of water being sampled. Furthermore, a comparison of calculated pH_tot_ from samples of DIC, TA and nutrients simultaneously collected from the surface sampling system and from inside one of the control chambers with a Niskin bottle, agreed to within pH 0.001. This indicates that the carbonate chemistry of the seawater as it was pumped from the chambers to the surface sensor array did not change substantially and suggests that much of the difference between the ambient pH and the pH measured in the antFOCE chambers was caused by greater variability in biological production inside the chambers themselves.

Observations of the seasonal variation in coastal carbonate chemistry in Antarctica are currently limited to four regions^20,23,24,39^. A seasonal cycle of carbonate chemistry in coastal Antarctica features pH values elevated by primary production in summer and lower values during winter driven by net heterotrophy. At McMurdo station in the Ross Sea for example, coastal pH ranges from 7.93 in June to 8.24 in January, with Ω_ar_ in January ranging from 1.79 to 2.03, while in June it reaches 1.03^23^. At Davis Research Station, East Antarctica, carbonate system parameters were measured between May and February, and the seasonal cycle of pH was found to vary from a low of 7.99 in September to 8.20 in January, while Ω_ar_ varied from 1.19 to 1.92 seasonally^20^. Seasonal ranges of pH observed at other Antarctic coastal sites vary from 0.27 at Ryder Bay^43^ and 0.53 at Palmer Station^24^ on the West Antarctic Peninsula; to 0.42 at McMurdo Sound^23^; to 0.21 at Davis in East Antarctica^20,38^.

This seasonal cycle is an important consideration for future FOCE experiments in polar regions, as a pH perturbation performed only in summer may not fully represent the extremes in carbonate chemistry that organisms will be exposed to under future atmospheric CO_2_ scenarios. For example, a −0.4 pH offset from ambient conditions maintained over a full year could result in pH levels as low as 7.53 in late winter. This is particularly relevant for Antarctic organisms that have life cycles across multiple seasons such as shelled pteropods^44^; invertebrates with sensitive larval stages^45,46^; or organisms with extremely long developmental periods^47^. Laboratory based ocean acidification experiments have shown adverse effects to the larval stages of Antarctic invertebrates at pH levels of 7.6–7.8^48–50^, which suggests that the timing of key life stages in relation to future changes in the seasonal cycle of carbonate chemistry will be important for an organism’s likelihood of survival. The weakly calcified shells (non-living) of Antarctic invertebrates have been shown to be vulnerable to dissolution, at a pH of 7.4 and Ω_ar_ of 0.47, within 14–35 days, with exposure of aragonitic or calcitic prisms in the inner shell architecture after 56 days^51^. Antarctic marine fauna have narrow physiological capabilities and are among those with the most limited capacity to cope with environmental change^46^, such as a very narrow window of tolerance to changes in temperature^52^. However, their capacity to cope with pH change is relatively poorly understood.

### Short-term ambient variability in carbonate chemistry

A comparison of observed ambient pH and dissolved oxygen concentrations at two separate locations within O’Brien Bay demonstrates the spatially variable nature of carbonate chemistry within Antarctic coastal ecosystems. The differences between the two sites are not only observed in the average daily carbonate parameters but are also apparent in the diel variability. The outer site had higher pH and dissolved oxygen concentrations (Figs 3, 4 and 6) and greater diel variability compared to the experiment site, particularly later in the summer (Figs 4 and 5). This variability was most likely driven by the different rates of biological productivity influenced by the different light regimes over each site. Thicker multi-year sea ice over the experiment site would have reduced the amount of light available for photosynthetic communities, compared to the thinner first-year ice over the outer site. The increased light availability at the outer site would have also exposed the area to greater diel variation in light, and therefore greater diel variability in biological productivity, particularly as the near 24-hour daylight at the start of the study period transitioned to day night cycles towards the end of summer. Further changes to light availability, such as variation in snow cover, may have also played a role. Differences in stratification or freshwater input are unlikely to be responsible for differences between sites, as a comparison of temperature and salinity between each site showed no significant differences (Figs 5 and 6) and salinity could explain similar amounts of variation in pH at both sites. This large degree of daily variation has also been observed under similar environmental conditions in McMurdo Sound in the Ross Sea^23,40^.

Estimates of primary production indicate it was highly variable, switching from net heterotrophy to net production from day to day. Both carbon- and oxygen-based methods of estimating NCP agreed well with each other, which showed that each site, on average, was net autotrophic during the study period. However, given the large measurement uncertainties of our calculated DIC values, there are limitations in being able to accurately resolve the processes responsible for relatively small changes over short timescales. The calculated average NCP (2 and 3 mmol C m^−2^ day^−1^) of this study is somewhat less than the values observed in summer (December to February) at other Antarctic coastal sites; for example NCP in Ryder Bay on the West Antarctic Peninsula had an average value of 28 mmol C m^−2^ day^43,53^ and NCP in Prydz Bay, East Antarctica^20^, had an average value of 17 mmol C m^−2^ day^−1^. However, the higher sampling frequency of this study showed that daily NCP values could be as high as 131 mmol C m^−2^ day^−1^ and as low as −142 mmol C m^−2^ day^−1^.

It is apparent that natural variability over small localised spatial scales needs to be better characterised to understand drivers of carbonate chemistry in Antarctic coastal waters to aid in assessment of potential future effects of ocean acidification. Such information will also greatly assist in planning and interpreting manipulative field experiments like FOCE. Monitoring of ambient conditions revealed local variations in pH that were likely to be driven by differences in biological productivity (as measured by dissolved oxygen concentration) and salinity. There was a strong relationship between NCP (as indicated by dissolved oxygen) and pH observed in distanced-based linear models where oxygen could explain between 37 and 47% of the variation in pH. Light plays an important role in determining community composition in polar coastal waters^54,55^. Increases in available light on the seabed via changes in sea ice patterns may also have important influences on variability of pH in Antarctic coastal ecosystems through increased primary production and CO_2_ drawdown. Changes in sea ice cover and increasing atmospheric CO_2_ concentrations may synergistically affect benthic communities, promoting the growth of macroalgae in habitats under ice currently dominated by invertebrates.

Salinity had an even stronger correlation with pH at both sites, explaining between 53 and 80% of pH variation. This is likely to be due to inputs of freshwater from glacial ice and snow melt, which are also predicted to increase under future climate change scenarios^56^. Understanding drivers of local variability and the natural range of pH that organisms are already subjected to is important in manipulative pH experiments and will improve our understanding of the effects of ocean acidification across a wide variety of marine ecosystems.

In summary, this is the first *in-situ* experiment in a polar region to successfully create predicted ocean acidification conditions using CO_2_ enrichment in a controlled way. The FOCE *in-situ* system provides means to address some of the critical gaps in ocean acidification research^16^ such as full community representation (including natural, undisturbed microbial communities), under natural conditions of food availability, light and carbonate system parameters, without hydrodynamic isolation. FOCE represents an important complementary method to established techniques using laboratory based experiments, mesocosms or natural analogues such as CO_2_ vents^57,58^.

## Methods

**Figure 8:**
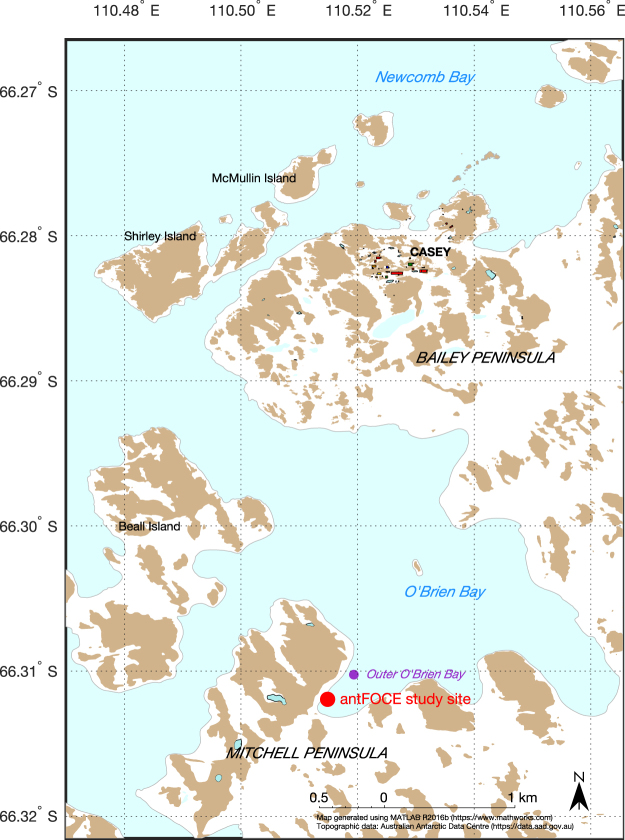
Map showing the location of the antFOCE experiment at Casey Research Station in East Antarctica. The experiment was conducted in a small bay within O’Brien Bay, between the Mitchell and Bailey Peninsulas in the Windmill Islands. Red dot shows location of the antFOCE experiment and location of the SeapHOx unit near the experiment; the purple marker for outer O’Brien Bay shows the location of the second SeapHOx unit. Map made using the mapping toolbox in MATLAB R2016b (https://www.mathworks.com). The topographic data was obtained from the Australian Antarctic Data Centre (https://data.aad.gov.au/aadc/portal/drill_down.cfm?id=14) and used with permission under the Creative Commons Attribution 3.0 Unported License.

**Figure 9:**
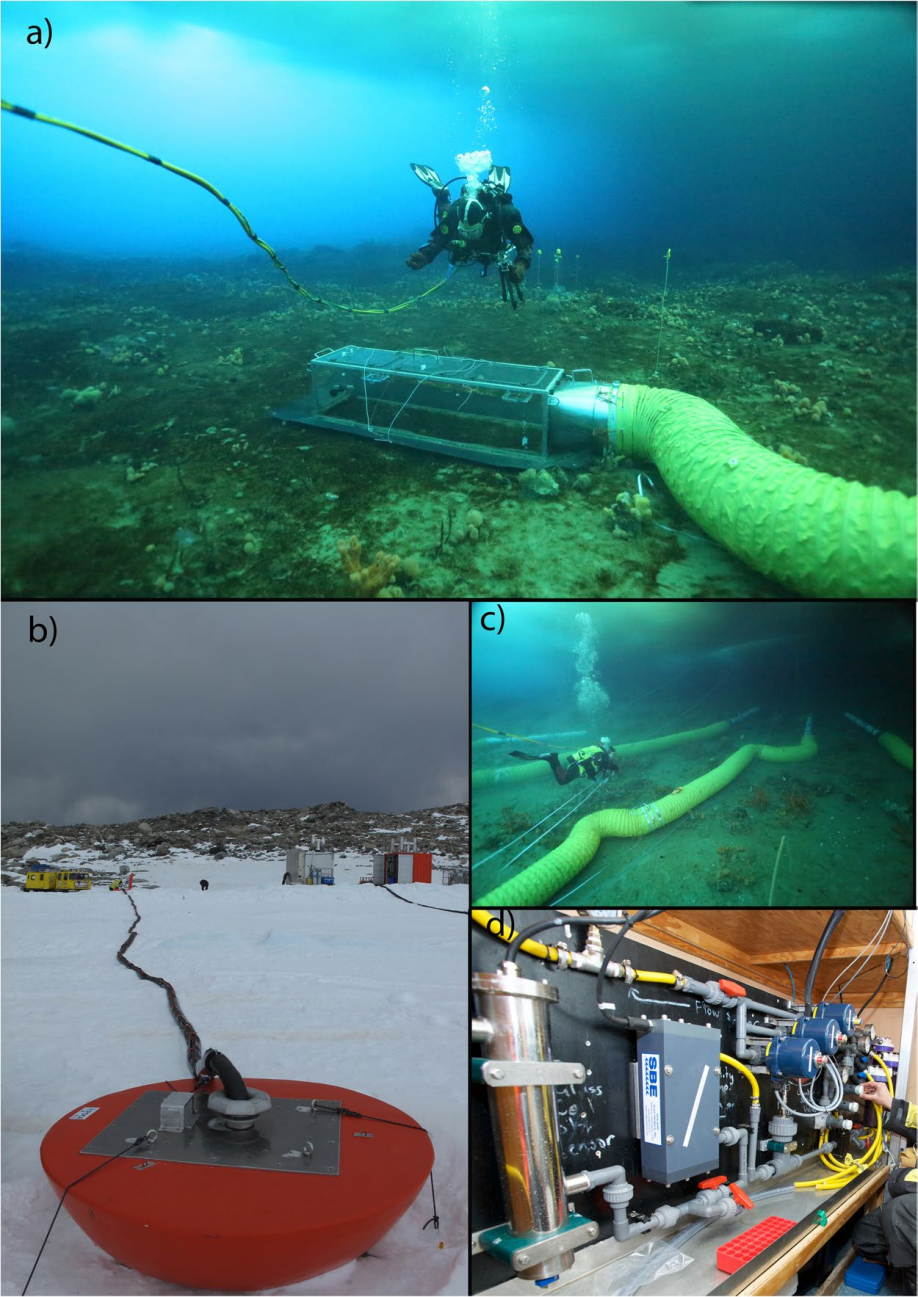
Photographs of the antFOCE system. (**a**) A diver over one of the experimental chambers, which is attached to an equilibration duct (Photo: J. Stark); (**b**) surface equipment at the antFOCE field site, with the sea-ice buoy and insulated umbilical in foreground, and the ‘Silver Chalet’, containing the surface sensor array, and orange container housing the generator and CO_2_ ESW system visible in the background (Photo: J. Stark); (**c**) the equilibration ducting with white thruster tubes attached (Photo: J. Stark); (**d**) the surface sensor array inside the Silver Chalet (Photo: N. Roden).

The antFOCE system was deployed in a small sheltered embayment within the larger O’Brien Bay (Fig. 8) in December 2014 after divers had surveyed for suitable areas to locate the experimental setup. The site was selected based on the following criteria: 1) accessibility from shore, 2) shallow water depth (for convenient diver access), 3) a mixed habitat of soft sediment and small boulders that supported a diverse and abundant invertebrate community, and 4) absence of contamination from Casey Research Station. The antFOCE experiment consisted of two replicate acidified chambers (chambers A and B) and two replicate control chambers (chambers C and D), which enclosed sections of seabed; and two open control plots (no chambers, plots E and F), at a depth of ~13 m beneath sea ice. The ice was approximately 260 cm thick at the start of the experiment and 200 cm thick by the end. The pH within the acidified chambers was manipulated by the incremental addition/adjustment of CO_2_ enriched seawater (ESW). This was controlled and monitored by a surface sensor array and a series of adjustable pumps and switchable taps (Fig. 9d) that also allowed seawater to be sampled from the surface. The chambers were placed on patches of sediment (Fig. 9a) at least 10 cm deep and at least 9 m^2^ in extent so that sediment/water interactions could be monitored. The benthic habitat was a mixture of boulders, cobbles and patches of sediment (Fig. 9a) and was dominated by marine invertebrates, with no macroalgae present due to the multi-year sea ice reducing light availability. The sediment was covered with a layer of microphytobenthos, consisting primarily of diatoms^59^. Divers deployed the underwater components over a period of several weeks while the surface system was transported to the site and assembled. A schematic diagram of the system is shown in Supplementary Fig. S4.

### CO_2_ Enriched Seawater (ESW)

Seawater from near the seabed, close to the experiment site, was pumped to the surface and saturated with CO_2_ in a pressurized cylinder (internal measurements 1520 mm long × diameter 240 mm, internal volume 69 L), which contained a roughly equal mix of air and CO_2_, held at approximately 1.6 atmospheres in order to ease the load on the dosing pump pushing ESW into the underwater chambers. The ESW system was housed in a shipping container with the generator. A mass flow controller (Alicat M series) was used to measure the amount of CO_2_ being used, which was supplied from a bank of 69 CO_2_ cylinders (food grade F size cylinder, holding 22 kg of CO_2_) stored on site adjacent to the generator van. The ESW cylinder contained a misting showerhead at the top and was filled with plastic bio-balls (OVI-Flow ball 2 Biologic Media) to increase the surface area available for diffusion. The ESW, which had an approximate pH of 5.5 and a CO_2_-enrichment factor in excess of 30, was introduced to the seabed experimental setup via a pump and hose system, as discussed below.

The chemical equilibrium of FOCE experiments is dependent on a time delay following addition of ESW, due to the relationship between pH, CO_2_ concentration and temperature as defined by the CO_2_ kinetic model of e-folding time (τ)^29^. In FOCE experiments, equilibrium between the various carbonate species is assumed to be reached at 3 × τ^14^. Given seawater temperatures of approximately −1.2 °C and a flow rate of ~8 L min^−1^ within the equilibration ducting, it was determined that 40 metres of ducting would be required for full chemical equilibration of the ESW to occur before it reached the experimental chambers.

### Seabed experimental setup

The four chambers were constructed from clear polycarbonate material (Fig. 9a) that measured 2000 mm length × 500 mm width × 500 mm height, with three hinged lids on the top of the chamber and an open bottom. On the outlet end of the chamber there was a 10 mm stainless steel mesh screen. A 280 mm wide rubber skirt flap was attached to the base of the chamber to help seal the sides (Fig. 9a). The chamber was attached to a 40 m long cylindrical equilibration duct (Plastcorp Mine flex stainless with steel wire; 500 mm diameter) via a stainless steel collar (Fig. 9a,c). Each duct consisted of 4 × 10 m long sections joined with stainless steel collars. The inlet end of the duct was attached to 3.5 m of PVC storm water pipe, known as the thruster tube. Each thruster tube consisted of two sections of PVC pipe (250 mm diameter) joined with a collar. The ESW was introduced to the start of the pipe just behind the open end used for the intake of surrounding seawater, which was fitted with a cylindrical section of 10 mm stainless steel mesh (370 mm long, 250 mm diameter) to prevent the ingress of fish and large invertebrates. In the second section, a thruster (Navigator 65-5501) drew water into the pipe past a flow meter (General Oceanics digital lower flow) and into the duct and then eventually through the chamber. Water flow through the system was unidirectional and maintained at 3 cm s^−1^, based on measurements taken at the site which showed background currents to range from 1 to 2 cm.s^−1^. The decision to use a slightly higher velocity was made to minimize the settlement of water borne particulates in the 40 m mixing ducts.

### Pump system and hoses

Peristaltic pumps were used to: 1) pump ambient seawater from near the chambers to the ESW unit (1× Albin peristaltic ALP17 56 RPM fixed), 2) pump ESW down to the thruster tubes (2× Albin peristaltic ALP17 29-6 RPM variable output speed), and 3) pump water from the chambers to the sensor panel (3× Albin peristaltic ALP17 29 RPM fixed). These pumps were contained in a large insulated plastic box (Techni Ice 1100 L), which was heated internally with a diesel-burning heater (Webasto WEB902023480B). Pressure rated hoses were used to draw up water to use in the manufacture of ESW (PVC Plutone clear/wire suction internal diameter 19 mm) and to the sensor panel from the chambers (PVC Plutone clear/wire suction 16 mm), as well as carry ESW to the thruster tubes (Barfell Diver air, internal diameter 10 mm). The hoses were wrapped in a double coil of heat trace and all wrapped by a layer of insulation (FR Armaflex) contained within a canvas wrapping, to prevent seawater freezing in the sub-zero air temperatures (Fig. 9b). The umbilical of the hoses, which also contained a 240 V power cable, crossed the surface of the sea ice to a large metal sea-ice buoy, which sat in a 0.9 m diameter hole in the sea ice (Fig. 9b). The umbilical and cables passed through the centre of the buoy, into the water (Fig. 9b), and out to the seabed experimental setup. Power to the entire antFOCE system was supplied from a Cummins C22 D5 (X series) generator, housed on-site in a shipping container. An adjacent 400 L tank of diesel fuel was connected to the generator and regularly refilled from 200 L drums transported to the site.

### Sensor array

Water was continuously pumped from the outlet end of three of the benthic chambers (both acidified chambers and one control, manually alternating between chamber C and D) to the surface where it was pumped through the sensor array, which was housed in an insulated building mounted on a sled, known as the Silver Chalet, which also housed the computers and electronic control equipment (Fig. 9b and d). To obtain measurements, valves were used to enable water to flow sequentially from each chamber over the sensors for 10 minute periods. Water from chambers not being passed over the sensors went into an overflow disposal line. The sensor array consisted of a dissolved oxygen optode (Aanderaa, model 3835), thermosalinograph (Seabird SBE 45 MicroTSG), and for redundancy, two pH electrodes (Seabird SBE 18 and Honeywell Durafet). Water from the chambers was sampled for biogeochemical measurements using a valve on the water line leading into the sensor array. An additional pH electrode (Seabird SBE 18) was moved between chambers as a means to compare pH measurements *in-situ* with those from the surface sensor panel.

### Control of pH

The pH of the acidified chambers was controlled by varying the amount of ESW pumped into the thruster tubes/equilibration ducts to achieve the −0.4 pH offset. The pH was measured by the Honeywell Durafet sensor and the flow rate of the ESW was adjusted slowly by a feedback control loop to ensure stability. Due to the surface sensor system cycling through each chamber every 10 minutes, and the seawater itself taking ~10 minutes to be pumped from the chamber to the surface, a delay of 30 minutes between estimates of pH offset was introduced. Adjustments to pH in the acidified chambers would then take another 10 minutes to propagate back down the ESW line and a further 22 minutes to flow through the equilibration ducting and into the chambers, introducing a total delay of ~1 hour in adjusting the pH within a chamber. This is necessary to let the CO_2_ chemistry reach equilibrium and create a true pH-altered environment as opposed to a high CO_2_ partial pressure situation^29^.

### Biogeochemical measurements

Discrete **s**eawater samples of 250 mL, used for the analysis of DIC and TA, were collected from the surface sampling system and from a Niskin bottle at various locations within the study site. A preservative of 100 μL of a saturated HgCl_2_ solution was added to these samples to halt biological activity. DIC was determined using a Single Operator Multiparameter Metabolic Analyser (SOP 2)^60^, and TA was determined by open-cell potentiometric titration using a 0.1 M hydrochloric acid titrant (SOP 3b)^60^. Routine analysis of Certified Reference Material (batch 137) from Scripps Institution of Oceanography were used to verify the measurement accuracy and precision for DIC and TA analyses, which were better than ±1.0 μmol kg^−1^ and ±1.3 μmol kg^−1^ respectively. Dissolved nutrient concentrations for nitrate, nitrite, phosphate and silicic acid were determined using standard colorimetric methods^61^, adapted for flow injection analysis on a SEAL AutoAnalyzer 3 HR, and yielded a measurement precision of ±0.4 µmol kg^−1^, ±0.01 µmol kg^−1^, ±0.06 µmol kg^−1^ and 3 µmol kg^−1^, respectively.

The pH electrodes in the antFOCE system were calibrated using calculated pH_tot_ values (accuracy and precision pH ±0.005, based on propagated uncertainties of measured parameters), that were determined approximately every three days using measurements of total dissolved inorganic carbon (DIC), total alkalinity (TA) and nutrients from the discrete bottle seawater samples. Calculations were made using the standard set of carbonate system equations with the CO2SYS program^62^ and the dissociation constants of Roy, *et al*.^63^. Due to minimal drift in the Honeywell Durafet sensor with a stability better than pH ±0.005 over weeks to months^64^, a mean offset correction was applied based on all calibration samples. Significant sensor drift occurred in the Seabird SBE 18 electrodes, which required an alternative calibration procedure, whereby it was assumed that the sensor drift was linearly proportional in time. However, this procedure did not significantly improve the data quality. It was determined that the drift was variable in nature and not readily corrected. As such, only the Honeywell Durafet data are considered here.

Two SeapHOx units^64,65^ were deployed on the seafloor in O’Brien Bay to measure ambient pH, dissolved oxygen, temperature and salinity at 1 hour intervals from 16 December 2014 to 28 February 2015. The units were located in 13 m of water: one adjacent to the thruster tubes of the antFOCE system (experiment site) and used to characterize the ambient seawater conditions, and the other ~300 m further towards the mouth of O’Brien Bay (outer O’Brien Bay; Fig. 8) to asses variability within the bay. Honeywell Durafet pH sensors inside the SeapHOx units were calibrated using the approach described previously, by collecting samples next to the SeapHOx units with a Niskin bottle. Oxygen optodes (Aanderaa, model 3835) were calibrated at the Commonwealth Scientific and Industrial Research Organisation (CSIRO) Ocean Carbon Laboratory in Hobart, Tasmania, Australia before and after deployments using Winkler titrations. The calibration data were used to fit the optode response to a Stern-Volmer equation^66^, and yielded a relative accuracy of ±2 µmol kg^−1^. Data from the oxygen optode in the surface sensor array were affected by air bubbles trapped inside its housing and are not considered any further in this study. The oxygen concentration at saturation was determined from atmospheric pressure, seawater temperature and salinity measurements^67^. Temperature and salinity were measured using two SBE 37-SI MicroCAT conductivity and temperature recorders that were calibrated at the CSIRO Oceanographic Calibration Facility in Hobart, Tasmania, Australia, and yielded an accuracy of ±0.002 °C and ±0.004, respectively.

TA data from this study were combined with data from previous studies in the Southern Ocean that used the same measurement techniques, and a linear regression of salinity versus TA was calculated. This relationship (Fig. 2) was used to calculate TA from high-resolution salinity measurements. Data used to calculate the regression were from samples shallower than 500 m and south of 60°S and included measurements from the CO_2_/World Ocean Circulation Experiment (WOCE) hydrographic sections of the nearby Princess Elizabeth Trough in 2005, measurements from the southern end of the 1994 WOCE SR3 transect along 140°E, and measurements from the Baseline Research on Oceanography, Krill and the Environment – West (BROKE-West) study in 2006^68^. The correlation between the parameters in Fig. 2 (y = (62.8 ± 0.4)x + (163 ± 13); n = 340, r^2^ = 0.99, standard error = 4 μmol kg^−1^) indicates net calcification/dissolution of carbonate minerals in the water column was not a significant contributor to the TA variability. The saturation state of aragonite (Ω_ar_)^69^ and the fugacity of CO_2_ (*f*CO_2_) were calculated from high-resolution measurements of pH_tot_, calculated TA, and the mean phosphate and silicic acid concentrations measured throughout the experimental period of 1.8 ± 0.2 µmol kg^−1^ and 50 ± 9 µmol kg^−1^, respectively. Propagating the uncertainties associated with these measured and calculated input parameters, we estimate an uncertainty of ±0.01 for Ωar and ±8 μatm for *f*CO2. All carbonate chemistry data is available at 10.4225/15/5a28b5eb04f71.

The NCP estimates for each SeapHOx deployment were calculated using a mass balance approach by averaging the calculated DIC concentrations for each day and subtracting one day’s value from the previous to estimate at a total daily DIC change. This change is considered to be the sum of changes due to freshwater flux from ice formation/melt, biology and air-sea flux, and was calculated using established procedures^20^. The biological component of this change can therefore represent the net community production (where positive) or net community respiration (where negative) for an entire 24 hour period. Oxygen-based “Redfield” estimates of NCP were calculated by dividing the daily change in oxygen by 1.4 to allow expression in terms of organic carbon production, or DIC uptake^33^.

### Data analysis

A four factor permutational analysis of variance (PERMANOVA^70,71^) was used to examine the differences in pH at five time scales, with the factors Month (3 levels), Week (3 to 5 levels, nested in month), Day (7 levels, nested in week), Diel (AM/PM, nested in day) and Hour (replicate measurements, residual variation). The analysis was based on the Euclidean distance similarity matrix with Type III (partial) sums-of-squares calculated using 999 unrestricted permutations of the raw data. PERMANOVA was also used to estimate the components of variation, a measure of the magnitude of effect size, for each temporal scale. Distance-based linear modelling of the relationship between pH and salinity, temperature and oxygen concentration were explored using the DISTLM procedure^35,71^, based on a Euclidean distance similarity matrix for pH and normalised salinity, temperature and dissolved oxygen.

## Electronic supplementary material

Supplementary Information
