# Supplementary material for: Carbonate chemistry of an in-situ free-ocean CO2 enrichment experiment (antFOCE) in comparison to short term variation in Antarctic coastal waters
Source: Sci Rep. 2018 Feb 12;8:2816. doi: 10.1038/s41598-018-21029-1 (PMC5809532; doi:10.1038/s41598-018-21029-1)
Supplement: Supplementary file 1 — Supplementary Information [file 41598_2018_21029_MOESM1_ESM.pdf]

Supplementary Information for: Carbonate chemistry of an in situ free-ocean CO_2 enrichment experiment (antFOCE) in comparison to short term variation in Antarctic coastal waters

J.S Stark, N.P. Roden, G. Johnstone, M. Milnes, J. Black, S. Whiteside, W. Kirkwood, K. Newbery, S. Stark, E. van Ooijen, B. Tilbrook, E.T. Peltzer, K. Berry, D. Roberts

Supplementary Table S1: Summary of weekly mean carbonate chemistry conditions in antFOCE experiment showing pH_{tot} , aragonite saturation state and fugacity of fCO_2. Data are shown for the acclimatization period (19 December 2014 – 2 January 2015) and experimental period (2 January 2015 – 27 February 2015), with the total number of downtime days (system stoppages) for each corresponding week and period at the bottom of each column. Ambient seawater data are from experiment site. s.d. = standard deviation. Offset shows the difference between the ambient and control chambers, and acidified and control chambers.

	Acclimatization period			Experimental period								
	Week 1	Week 2	Mean	Week 1	Week 2	Week 3	Week 4	Week 5	Week 6	Week 7	Week 8	Mean
	mean pH_{tot} (± 1 s.d.)											
Ambient seawater	8.054 ± 0.005	8.049 ± 0.009	8.052 ± 0.008	8.043 ± 0.008	8.053 ± 0.006	8.065 ± 0.011	8.084 ± 0.010	8.091 ± 0.007	8.101 ± 0.015	8.102 ± 0.008	8.108 ± 0.008	8.081 ± 0.025
Control chambers	7.997 ± 0.019	8.005 ± 0.037	8.001 ± 0.029	8.036 ± 0.032	8.023 ± 0.041	8.055 ± 0.031	8.067 ± 0.026	8.082 ± 0.030	8.099 ± 0.033	8.099 ± 0.035	8.100 ± 0.032	8.068 ± 0.044
$Offset_{cont-amb}$	-0.059 ± 0.020	-0.049 ± 0.037	-0.054 ± 0.030	-0.006 ± 0.035	-0.029 ± 0.040	-0.010 ± 0.031	-0.015 ± 0.028	-0.009 ± 0.031	-0.003 ± 0.032	-0.004 ± 0.032	-0.009 ± 0.030	-0.011 ± 0.034
Experiment chamber (A)	7.978 ± 0.060	7.948 ± 0.057	7.964 ± 0.060	7.749 ± 0.110	7.645 ± 0.091	7.660 ± 0.050	7.682 ± 0.102	7.683 ± 0.063	7.705 ± 0.062	7.701 ± 0.081	7.700 ± 0.072	7.687 ± 0.085
$Offset_{exp-cont}$ chamber (A)	-0.020 ± 0.058	-0.059 ± 0.067	-0.038 ± 0.065	-0.290 ± 0.113	-0.381 ± 0.057	-0.395 ± 0.028	-0.391 ± 0.074	-0.402 ± 0.025	-0.392 ± 0.054	-0.405 ± 0.034	-0.402 ± 0.042	-0.383 ± 0.066
Experiment chamber (B)	7.981 ± 0.053	7.945 ± 0.051	7.964 ± 0.055	7.752 ± 0.105	7.654 ± 0.088	7.655 ± 0.050	7.688 ± 0.097	7.682 ± 0.051	7.701 ± 0.051	7.701 ± 0.073	7.697 ± 0.060	7.688 ± 0.078
$Offset_{exp-cont}$ chamber (B)	-0.017 ± 0.053	-0.059 ± 0.056	-0.036 ± 0.058	-0.285 ± 0.109	-0.370 ± 0.056	-0.400 ± 0.027	-0.382 ± 0.076	-0.400 ± 0.026	-0.399 ± 0.032	-0.401 ± 0.041	-0.404 ± 0.033	-0.382 ± 0.065
	mean aragonite saturation state (Ω_{ar}) (± 1 s.d.)											
Ambient seawater	1.36 ± 0.01	1.36 ± 0.02	1.36 ± 0.02	1.35 ± 0.02	1.38 ± 0.02	1.42 ± 0.04	1.46 ± 0.04	1.43 ± 0.02	1.44 ± 0.03	1.44 ± 0.02	1.46 ± 0.03	1.42 ± 0.05
Control chambers	1.21 ± 0.05	1.24 ± 0.10	1.22 ± 0.08	1.33 ± 0.09	1.30 ± 0.11	1.39 ± 0.09	1.39 ± 0.07	1.40 ± 0.09	1.43 ± 0.09	1.43 ± 0.10	1.44 ± 0.10	1.39 ± 0.11
$Offset_{cont-amb}$	-0.16 ± 0.05	-0.13 ± 0.10	-0.15 ± 0.08	-0.02 ± 0.10	-0.08 ± 0.11	-0.03 ± 0.09	-0.04 ± 0.08	-0.03 ± 0.09	-0.01 ± 0.09	-0.01 ± 0.10	-0.03 ± 0.09	-0.03 ± 0.10
Experiment chamber (A)	1.17 ± 0.14	1.10 ± 0.13	1.14 ± 0.14	0.74 ± 0.19	0.58 ± 0.15	0.60 ± 0.07	0.62 ± 0.18	0.60 ± 0.11	0.62 ± 0.11	0.62 ± 0.15	0.62 ± 0.14	0.62 ± 0.15
$Offset_{exp-cont}$ chamber (A)	-0.04 ± 0.13	-0.14 ± 0.16	-0.09 ± 0.15	-0.60 ± 0.21	-0.72 ± 0.09	-0.79 ± 0.05	-0.78 ± 0.14	-0.81 ± 0.05	-0.80 ± 0.11	-0.82 ± 0.07	-0.82 ± 0.10	-0.77 ± 0.13
Experiment chamber (B)	1.17 ± 0.11	1.09 ± 0.12	1.14 ± 0.12	0.75 ± 0.18	0.60 ± 0.14	0.59 ± 0.07	0.63 ± 0.19	0.60 ± 0.08	0.61 ± 0.09	0.62 ± 0.13	0.61 ± 0.11	0.62 ± 0.13
$Offset_{exp-cont}$ chamber (B)	-0.04 ± 0.10	-0.14 ± 0.13	-0.08 ± 0.13	-0.59 ± 0.2	-0.70 ± 0.09	-0.80 ± 0.05	-0.77 ± 0.14	-0.81 ± 0.05	-0.82 ± 0.08	-0.82 ± 0.09	-0.83 ± 0.08	-0.77 ± 0.13
	mean fugacity of carbon dioxide ($f\ CO_2$) (µatm) (± 1 s.d.)											

Ambient seawater	368 ± 4	374 ± 9	371 ± 8	379 ± 7	370 ± 6	359 ± 10	340 ± 9	329 ± 6	319 ± 13	318 ± 7	314 ± 7	341 ± 25
Control chambers	425 ± 20	419 ± 37	422 ± 29	387 ± 31	400 ± 39	369 ± 29	354 ± 23	338 ± 26	321 ± 28	321 ± 28	321 ± 26	354 ± 42
$Offset_{cont-amb}$	59 ± 20	49 ± 38	55 ± 30	7 ± 34	30 ± 39	10 ± 29	13 ± 24	8 ± 26	3 ± 26	4 ± 26	8 ± 24	11 ± 31
Experiment chamber (A)	450 ± 75	485 ± 69	466 ± 74	810 ± 203	1028 ± 189	979 ± 110	938 ± 155	908 ± 112	855 ± 106	868 ± 129	868 ± 111	914 ± 159
$Offset_{exp-cont}$ chamber (A)	26 ± 74	69 ± 80	46 ± 79	428 ± 202	631 ± 149	610 ± 85	589 ± 127	575 ± 74	530 ± 93	556 ± 79	550 ± 75	564 ± 130
Experiment chamber (B)	446 ± 84	487 ± 62	465 ± 77	801 ± 191	1003 ± 179	990 ± 112	919 ± 153	908 ± 97	861 ± 92	865 ± 121	872 ± 100	911 ± 150
$Offset_{exp-cont}$ chamber (B)	23 ± 87	67 ± 66	43 ± 81	417 ± 190	605 ± 141	622 ± 86	569 ± 128	571 ± 69	541 ± 66	549 ± 86	553 ± 67	559 ± 124
Downtime (total days)	4.15	4.4	8.55	2.26	0.44	0.22	2.03	1.49	1.1	2.32	2.33	12.19

Supplementary Table S2: Summary of changes in ambient seawater conditions within O'Brien Bay, the site of the antFOCE experiment. a) daily changes and b) seasonal values between 19 December 2014 – 27 February 2015. O_2 = dissolved oxygen; DIC = calculated dissolved inorganic carbon; Ω_{ar} = saturation state of aragonite; fCO_2 = fugacity of CO_2.

**a) Daily changes**	**pH_{tot}**	**O_2 (µmol kg^{-1})**	**DIC (µmol kg^{-1})**	**Ω_{ar}**	**fCO_2 (µatm)**	**Salinity**	**Temp (°C)**
**Ambient (experiment site)**							
Minimum range	0.008	3	3	0.02	8	0.007	0.008
Maximum range	0.049	23	22	0.16	44	0.226	0.301
Mean range (± 1 s.d.)	0.020 ± 0.008	8 ± 3	9 ± 4	0.06 ± 0.02	18 ± 7	0.050 ± 0.048	0.076 ± 0.069
**Ambient (outer O'Brien Bay)**							
Minimum range	0.007	4	2	0.02	6	0.004	0.012
Maximum range	0.073	33	28	0.24	52	0.221	0.343
Mean range (± 1 s.d.)	0.029 ± 0.016	13 ± 7	11 ± 6	0.09 ± 0.05	23 ± 12	0.046 ± 0.045	0.082 ± 0.067
**Control chambers**							
Minimum range	0.014	-	5	0.04	10	-	-
Maximum range	0.127	-	47	0.37	115	-	-
Mean range (± 1 s.d.)	0.077 ± 0.027	-	28 ± 10	0.22 ± 0.08	69 ± 25	-	-
**Experimental chamber (A)**							
Minimum range	0.039	-	12	0.04	43	-	-
Maximum range	0.535	-	167	1.13	891	-	-
Mean range (± 1 s.d.)	0.242 ± 0.17	-	73 ± 52	0.46 ± 0.36	390 ± 246	-	-
**Experimental chamber (B)**							
Minimum range	0.046	-	14	0.07	49	-	-
Maximum range	0.545	-	167	1.1	835	-	-
Mean range (± 1 s.d.)	0.231 ± 0.16	-	69 ± 49	0.43 ± 0.34	382 ± 233	-	-

**b) Seasonal values**	**pH_{tot}**	**O_2 (µmol kg^{-1})**	**DIC (µmol kg^{-1})**	**Ω_{ar}**	**fCO_2 (µatm)**	**Salinity**	**Temp (°C)**
**Ambient (experiment site)**							
Minimum	8.025	333	2105	1.3	291	33.079	-1.479
Maximum	8.137	374	2206	1.55	397	34.257	-0.609
Range	0.112	41	100	0.25	106	1.178	0.87
Mean (± 1 s.d.)	8.075 ± 0.025	348 ± 7	2166 ± 32	1.41 ± 0.05	348 ± 26	33.785 ± 0.430	-1.120 ± 0.242
**Ambient (outer O'Brien Bay)**							
Minimum	8.052	335	2089	1.37	249	33.088	-1.466
Maximum	8.198	400	2196	1.79	371	34.256	-0.639
Range	0.146	65	107	0.42	122	1.168	0.827
Mean (± 1 s.d.)	8.107 ± 0.028	356 ± 11	2153 ± 32	1.51 ± 0.07	320 ± 25	33.776 ± 0.432	-1.112 ± 0.243

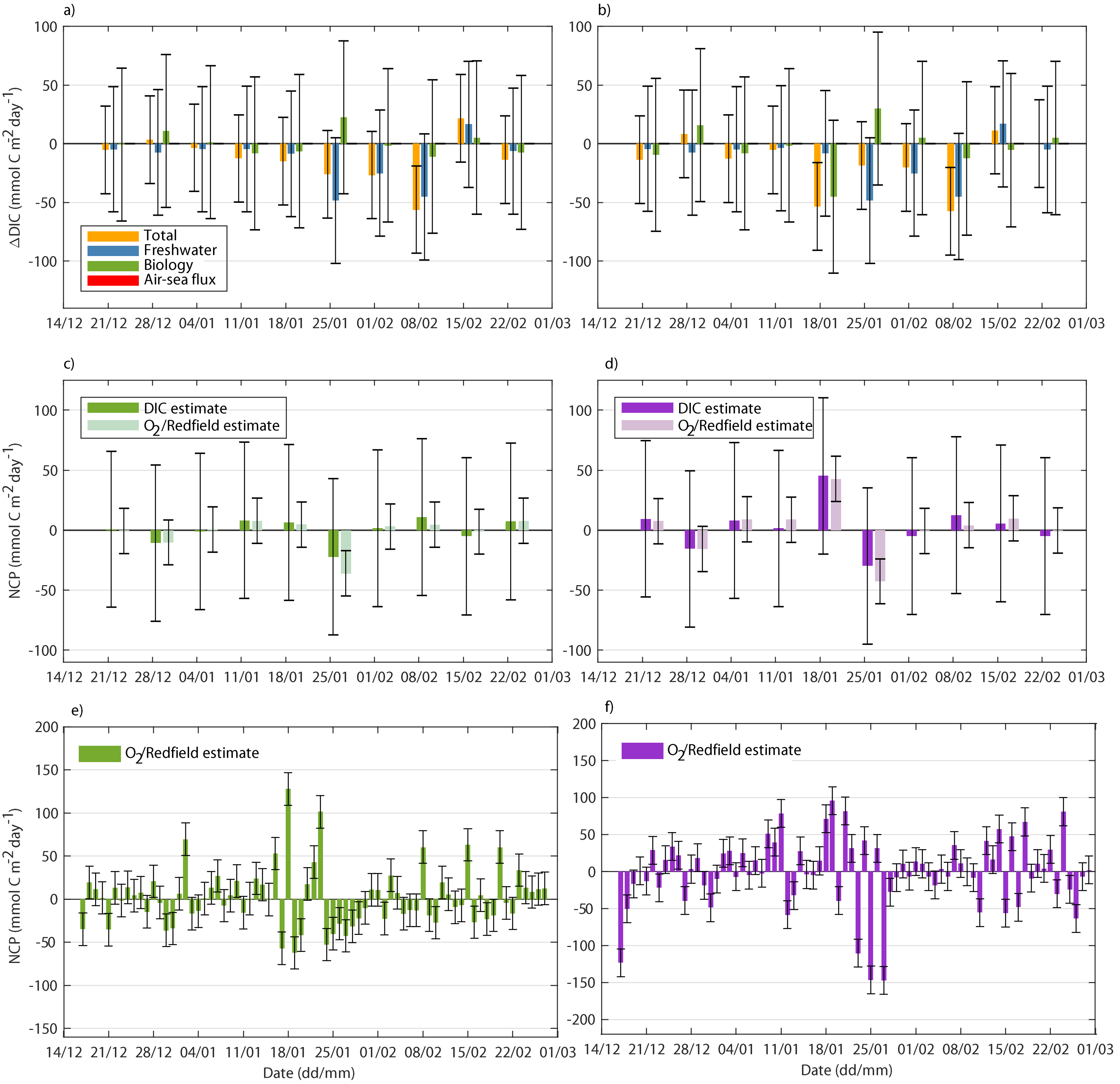

Supplementary Figure S3: Daily changes in ambient seawater conditions at two sites in O'Brien Bay, Casey station, Antarctica. At the experimental site, a) the weekly mean of daily changes in DIC partitioned into total change, and changes caused by freshwater input, biological activity and air-sea flux. c) The weekly mean of daily NCP values estimated from calculated DIC and measured oxygen and e) daily NCP estimates from calculated DIC. The corresponding parameters for the outer O'Brien Bay site are shown in panels b), d) and f) respectively. Error bars represent the propagated measurement uncertainties and positive NCP values indicate net primary production.

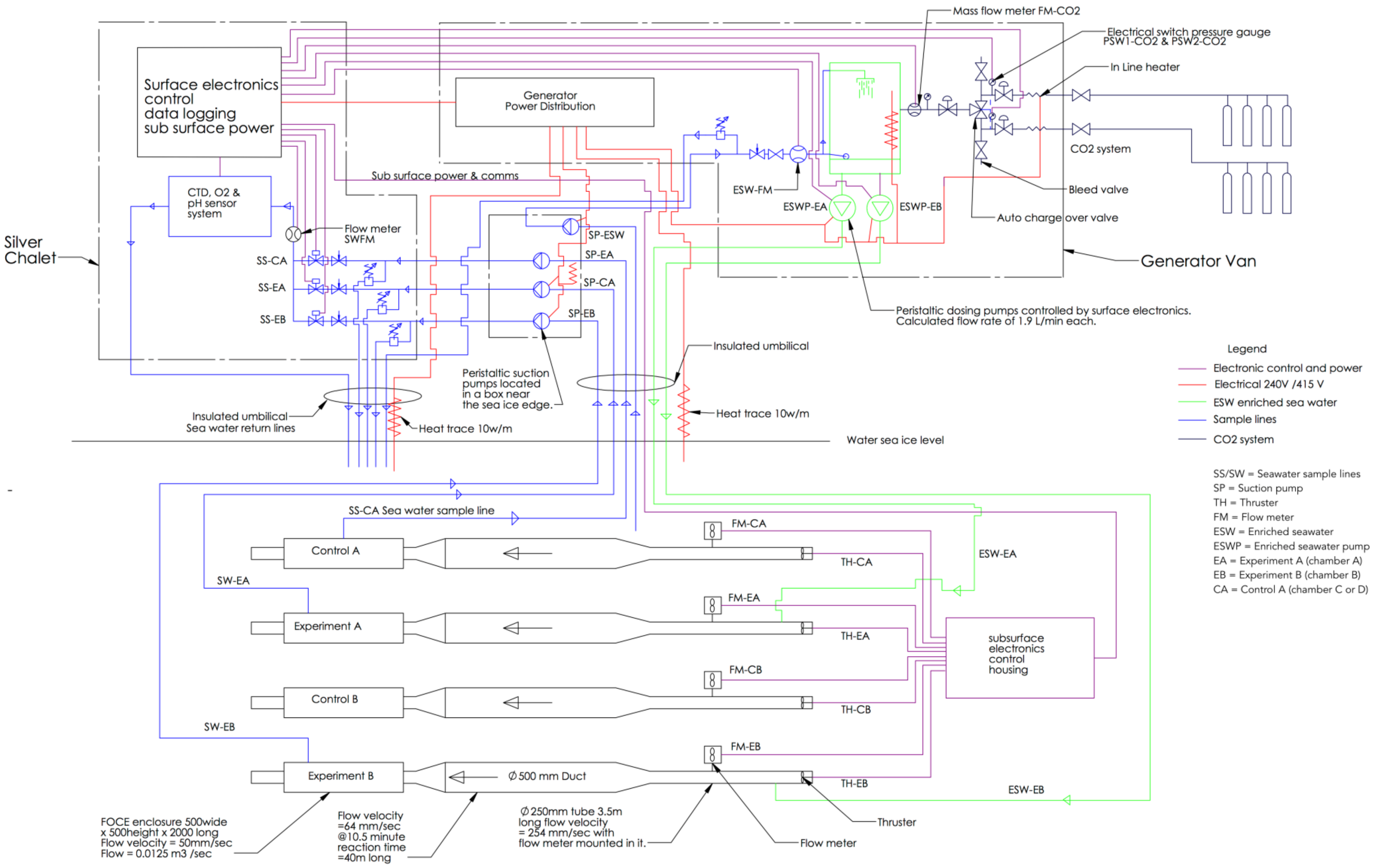

Supplementary Figure S4 Schematic plan of the antFOCE system components.
